# Supplementary material for: Predictive biomarkers for 5-fluorouracil and oxaliplatin-based chemotherapy in gastric cancers via profiling of patient-derived xenografts
Source: Nat Commun. 2021 Aug 10;12:4840. doi: 10.1038/s41467-021-25122-4 (PMC8355375; doi:10.1038/s41467-021-25122-4)
Supplement: Supplementary file 1 — Supplementary Information [file 41467_2021_25122_MOESM1_ESM.pdf]

Supplementary Figures & Tables

Predictive biomarkers for 5-fluorouracil and oxaliplatin-based chemotherapy in gastric cancers via profiling of patient-derived xenografts

Deukchae Na, Jeesoo Chae, Sung-Yup Cho, Wonyoung Kang, Ahra Lee, Seoyeon Min, Jinjoo Kang, Min Jung Kim, Jaeyong Choi, Woochan Lee, Dongjin Shin, Ahrum Min, Yu-Jin Kim, Kyung-Hun Lee, Tae-Yong Kim, Yun-Suhk Suh, Seong-Ho Kong, Hyuk-Joon Lee, Woo-Ho Kim, Hansoo Park, Seock-Ah Im, Han-Kwang Yang, Charles Lee, and Jong-Il Kim

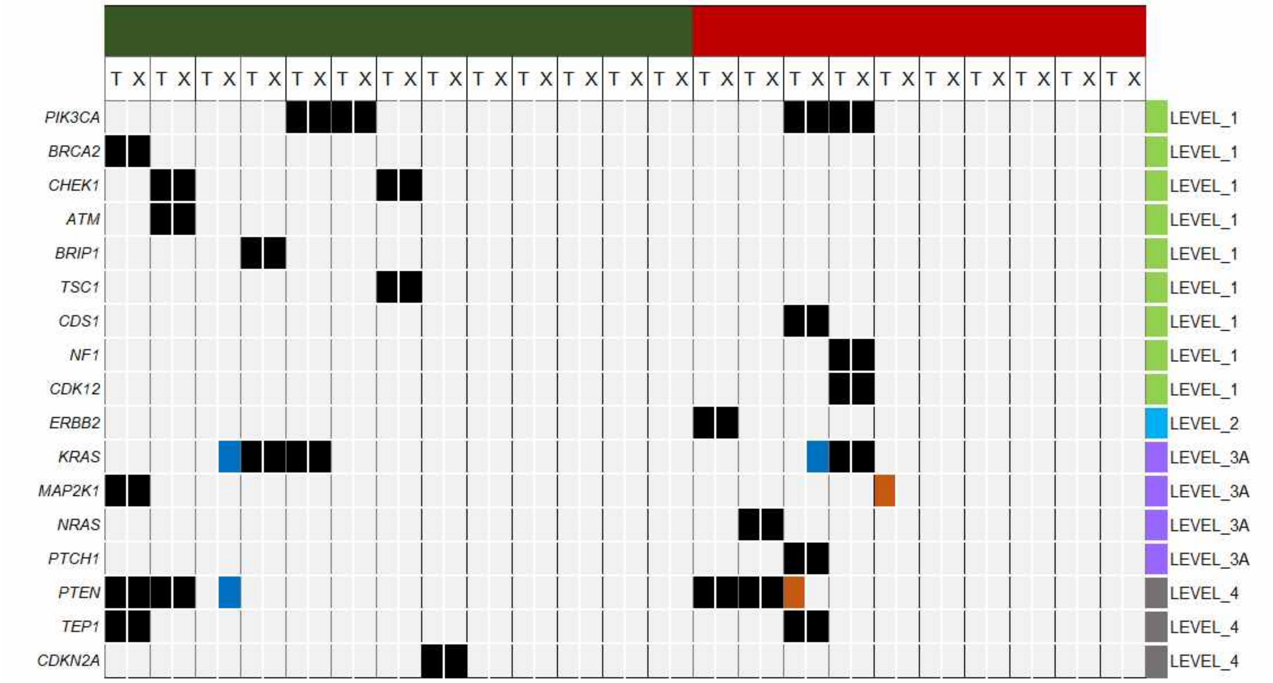

Supplementary Figure 1. Level 1-4 druggable somatic mutations on oncogenes annotated by OncoKB. Black: Both patient and PDX tumors harbor somatic mutations. Blue: PDX only. Orange: Patient tumor only. Level 1: FDA-approved drugs, Level 2: Standard care, Level 3: Clinical evidence, Level 4: Biological evidence.

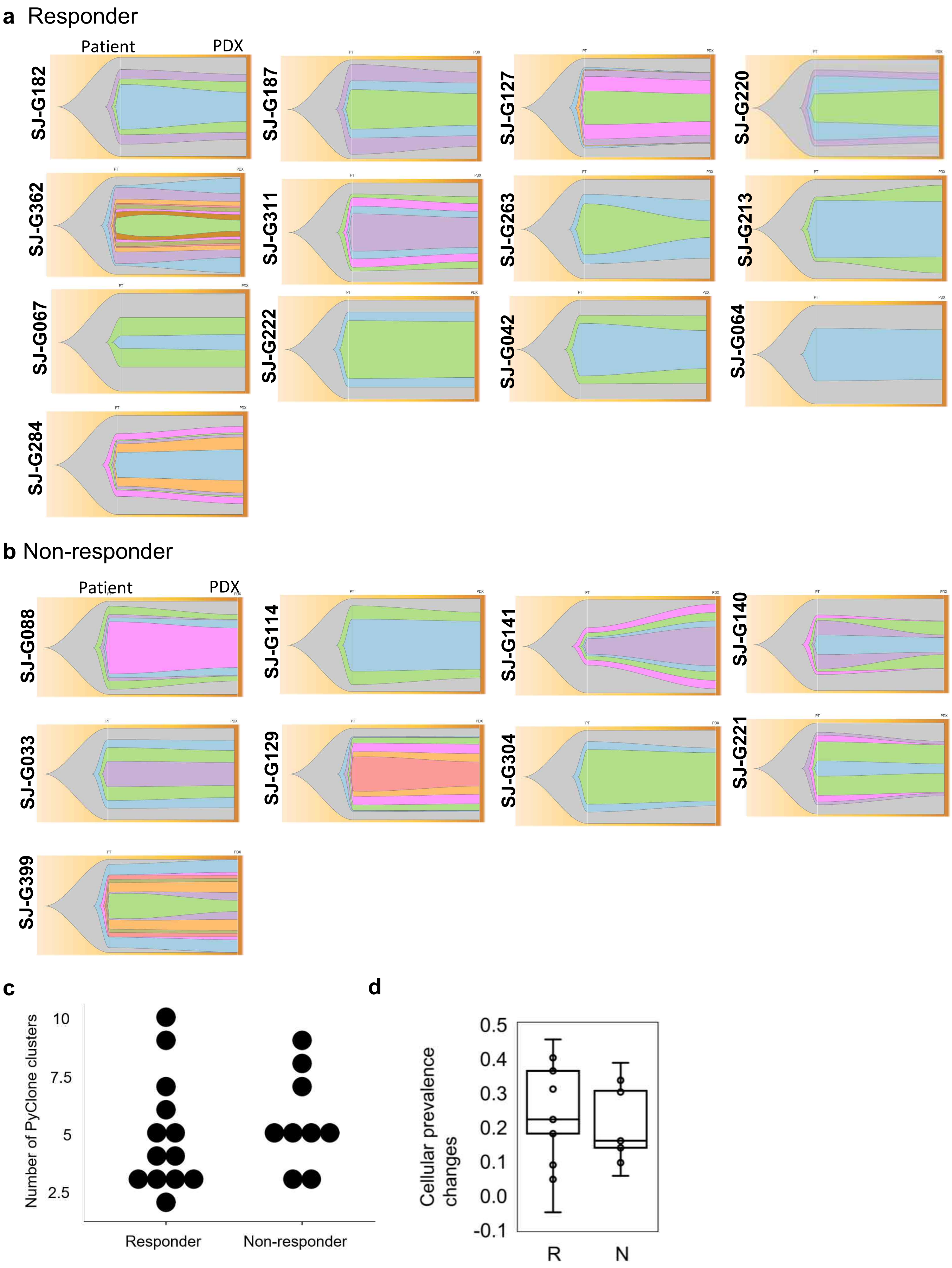

a Responder
Patient
PDX
SJ-G182
SJ-G187
SJ-G127
SJ-G220
SJ-G362
SJ-G311
SJ-G263
SJ-G213
SJ-G067
SJ-G222
SJ-G042
SJ-G064
SJ-G284
b Non-responder
Patient
PDX
SJ-G088
SJ-G114
SJ-G141
SJ-G140
SJ-G033
SJ-G129
SJ-G304
SJ-G221
SJ-G399
c
Number of PyClone clusters
10
7.5
5
2.5
Responder
Non-responder
d
Cellular prevalence changes
0.5
0.4
0.3
0.2
0.1
0.0
-0.1
R
N

Supplementary Figure 2. Clonal structure analysis using whole exome sequencing data. a, b, Changes in clonal structures between patient and PDX tumors inferred by PyClone in responder (n=13) and non-responder (n=9; excluding two samples with low number of mutations) groups. **c,** Number of Pyclone clusters (n=13 for responder, n=9 for non-responder). **d**, Cellular prevalence of changes between patient and PDXs in responder (R) and non-responder (NR) groups (n=13 for responder, n=9 for non-responder). Box plot were presented as median value and standard deviation. The bottom and top edges of the box indicate the 25th and 75th percentiles, respectively.

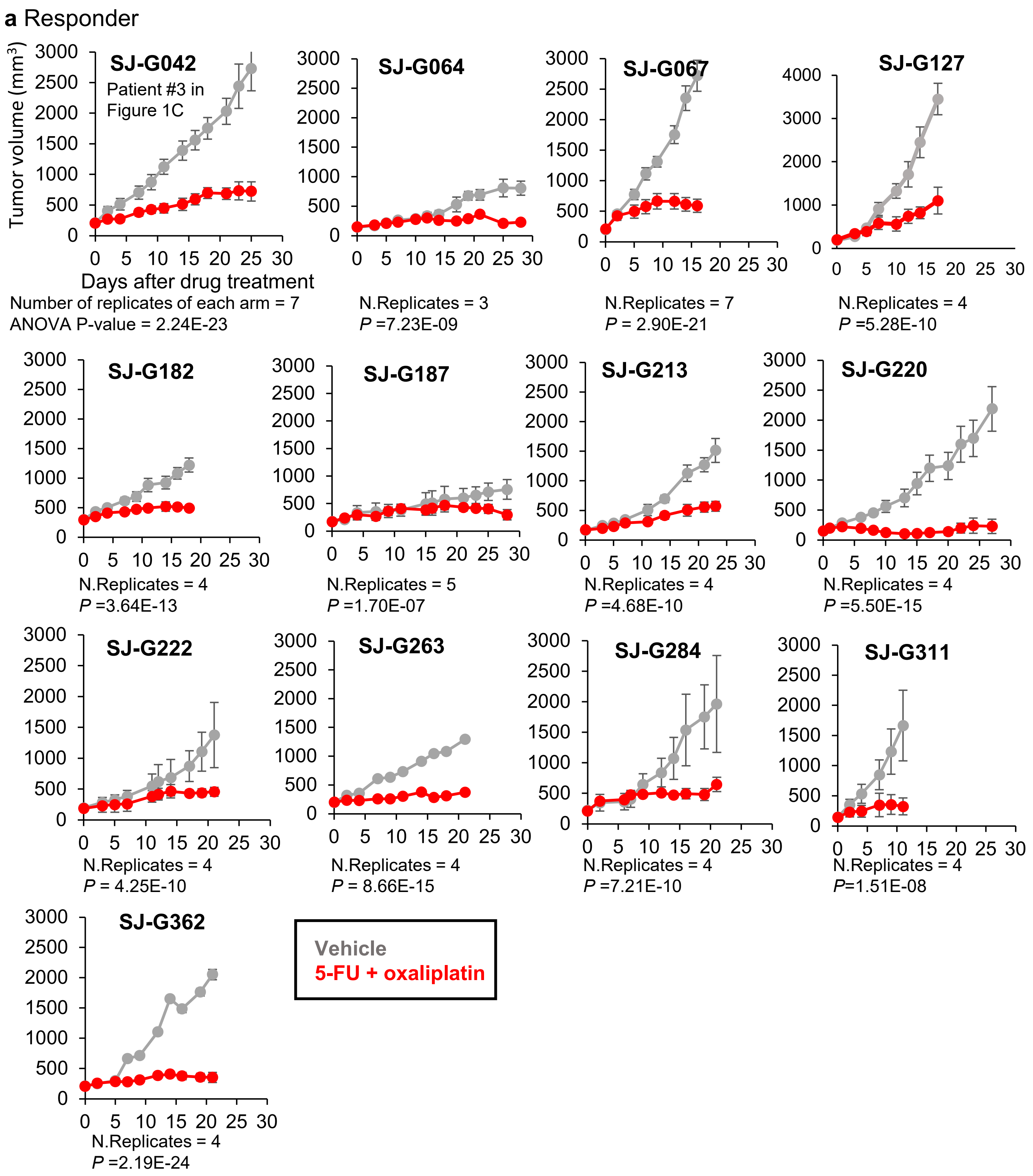

Supplementary Figure 3. 5-FU + oxaliplatin response screening test in 32 GC PDX models. a-c, Tumor growth curves of average volume of 5-FU + oxaliplatin treatment and vehicle groups are shown; a, responder, b, intermediate responder and c, non-responder. Data are presented as mean value and standard deviation. Two-way ANOVA with replication. Tumor growth observation data used in (a)-(c) were provided in Source Data.

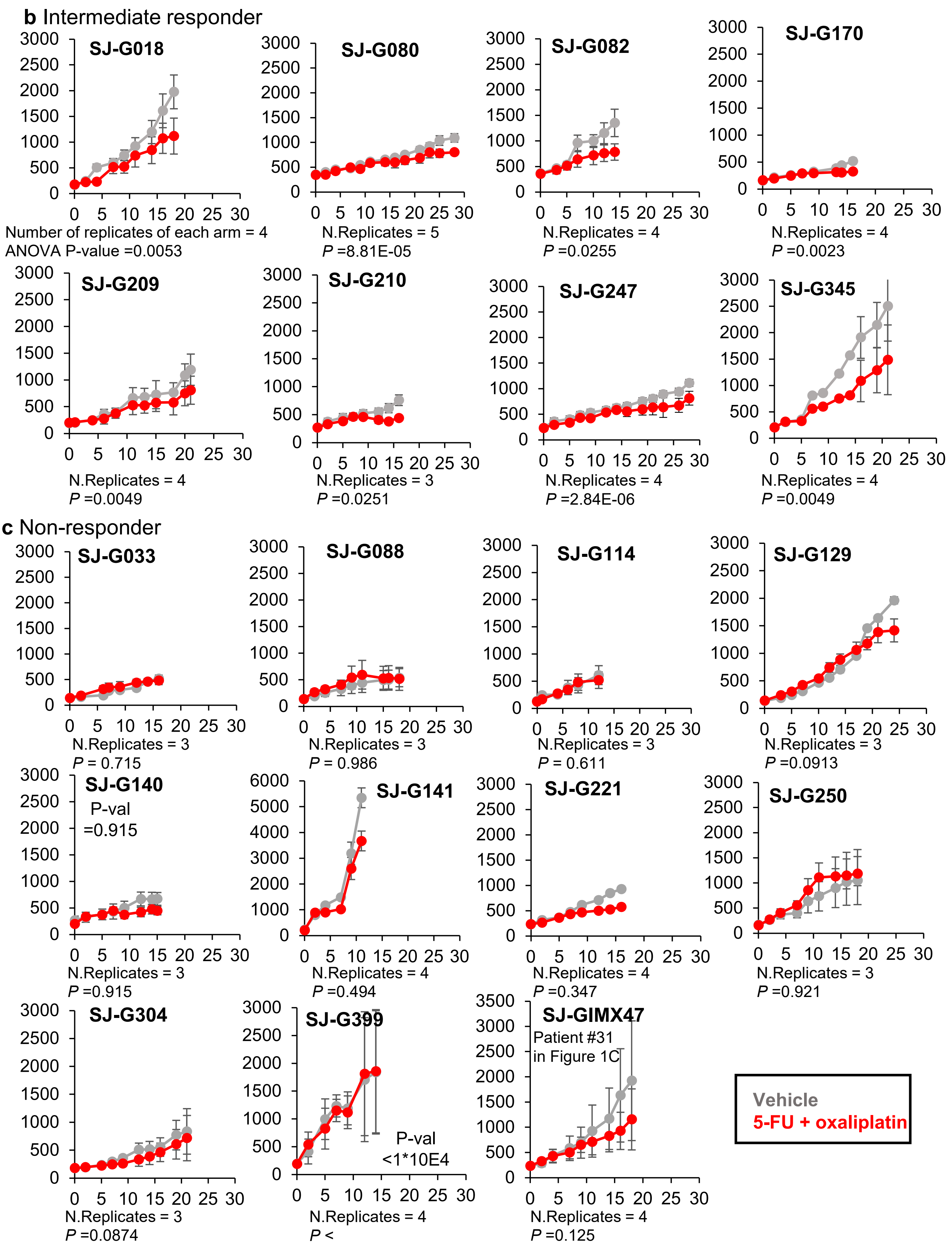

Supplementary Figure 3 (continued).

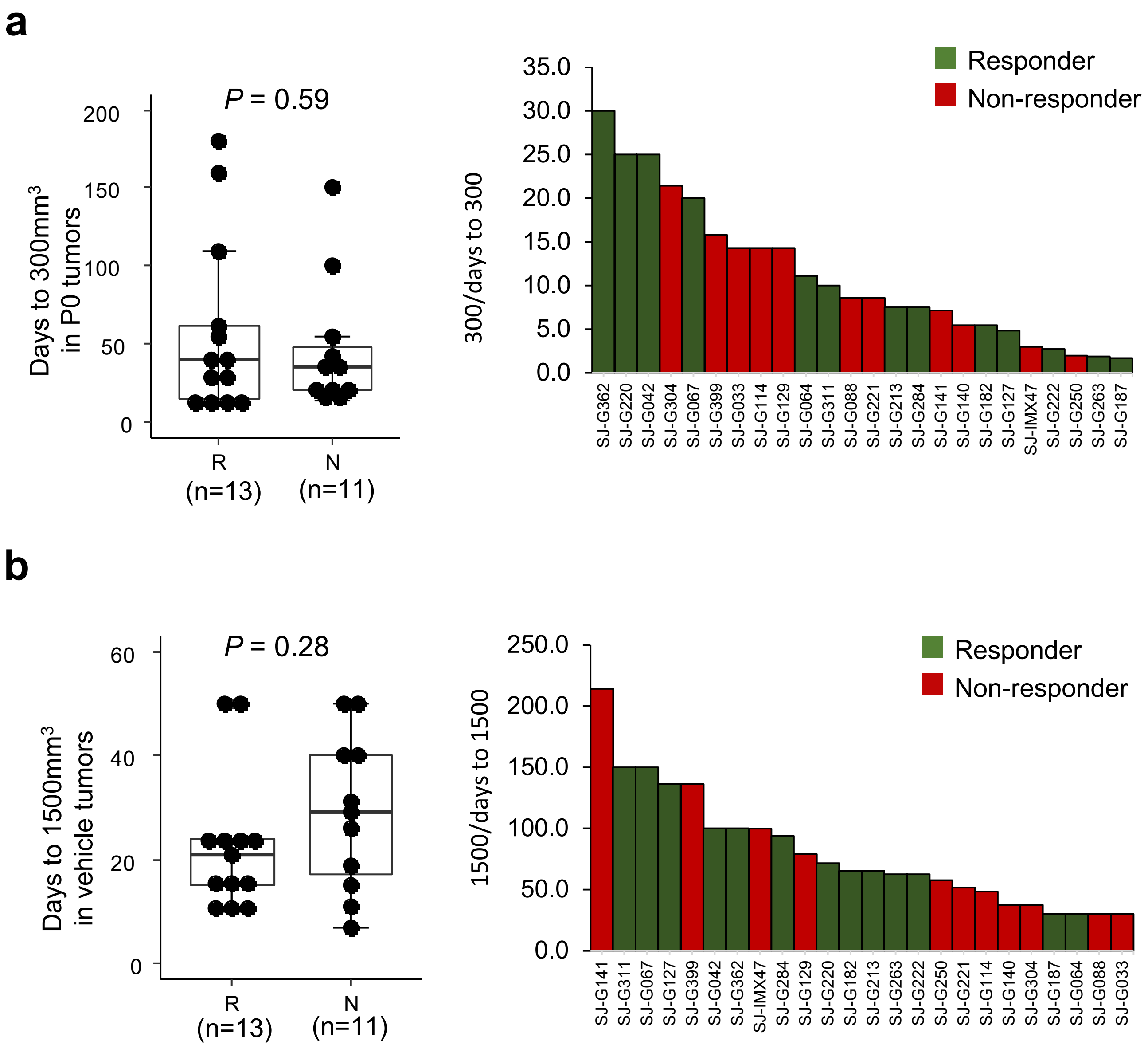

Supplementary Figure 4. Comparison of the number of days that tumor volume reaches a certain size. a,b, Number of days for tumor volume reaching 300 mm^3 during the patient-to-PDX engraftment (P0; a) and 1500 mm^3 in a vehicle group during screening test (b). Box plot were presented as median value and standard deviation. The bottom and top edges of the box indicate the 25th and 75th percentiles, respectively. Two-sided Student's T-test was performed. Tumor growth rate between the response and non-response groups was not significantly different.

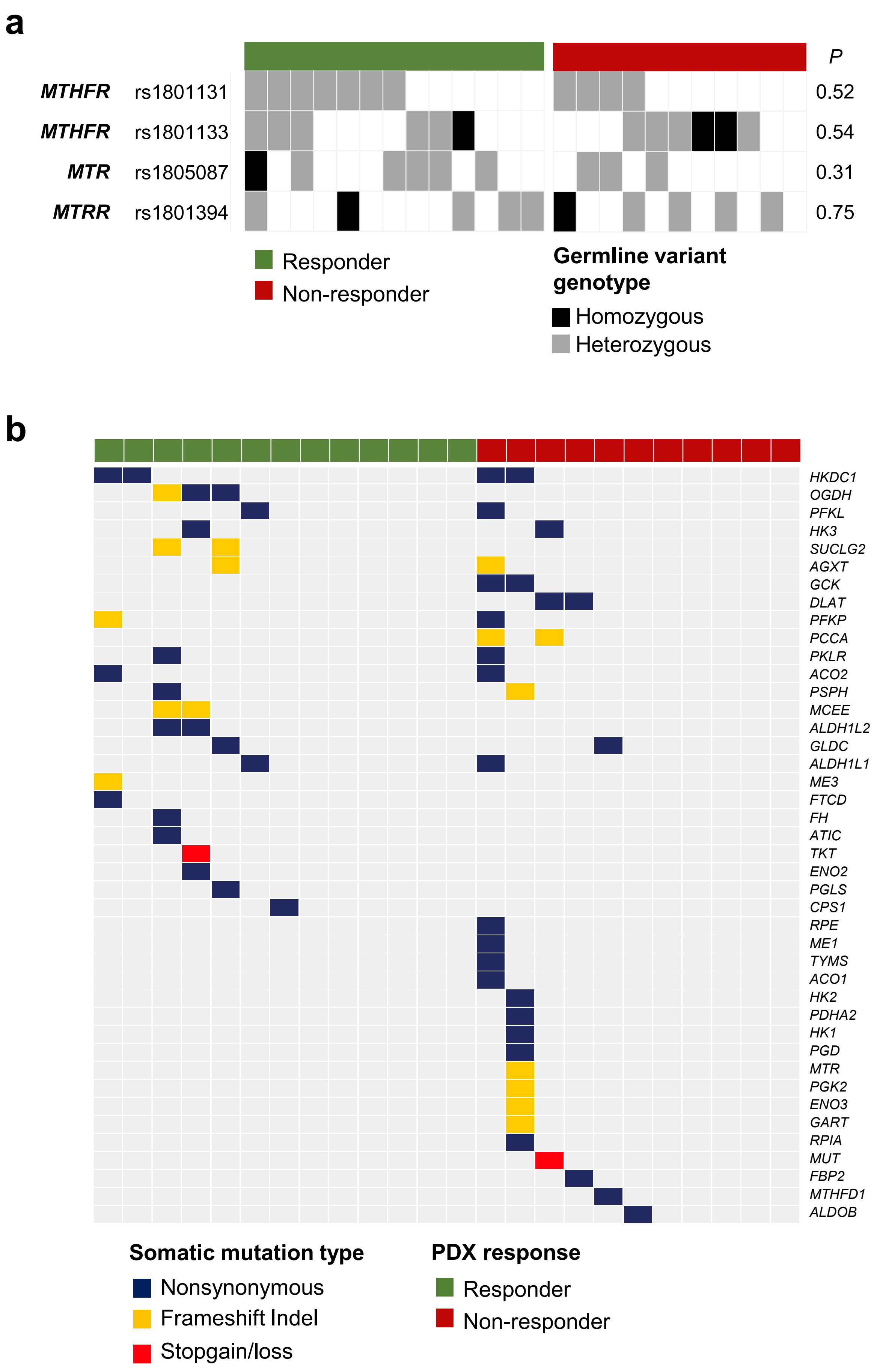
a
P
MTHFR rs1801131 0.52
MTHFR rs1801133 0.54
MTR rs1805087 0.31
MTRR rs1801394 0.75
Responder
Non-responder
Germline variant genotype
Homozygous
Heterozygous
b
HKDC1
OGDH
PFKL
HK3
SUCLG2
AGXT
GCK
DLAT
PFKP
PCCA
PKLR
ACO2
PSPH
MCEE
ALDH1L2
GLDC
ALDH1L1
ME3
FTCD
FH
ATIC
TKT
ENO2
PGLS
CPS1
RPE
ME1
TYMS
ACO1
HK2
PDHA2
HK1
PGD
MTR
PGK2
ENO3
GART
RPIA
MUT
FBP2
MTHFD1
ALDOB
Somatic mutation type
Nonsynonymous
Frameshift Indel
Stopgain/loss
PDX response
Responder
Non-responder

c

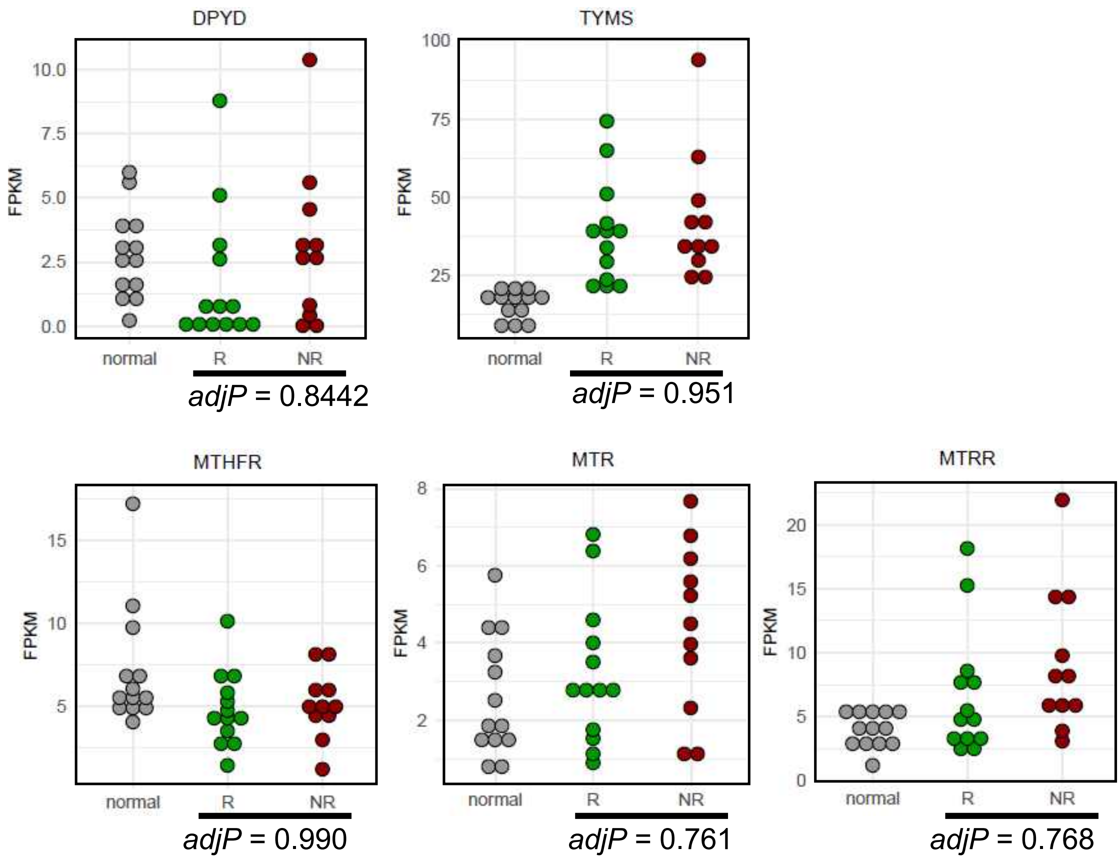

Supplementary Figure 5. Mutational profiles and dysregulation pattern of one-carbon metabolism (OCM) pathway. a, Germline variant status of previously reported genes and variants which might be related to 5-FU metabolism. Allele frequency of R and NR didn't show significant difference between two groups. Two-sided Fisher's exact test for P-values. **b**, Mutation profiles on carbon metabolism and one carbon pool by folate gene sets based on KEGG database. **c**, Gene expression of 5-FU metabolism associated genes was not significant. *P*-values attained by the Wald test corrected for multiple testing.

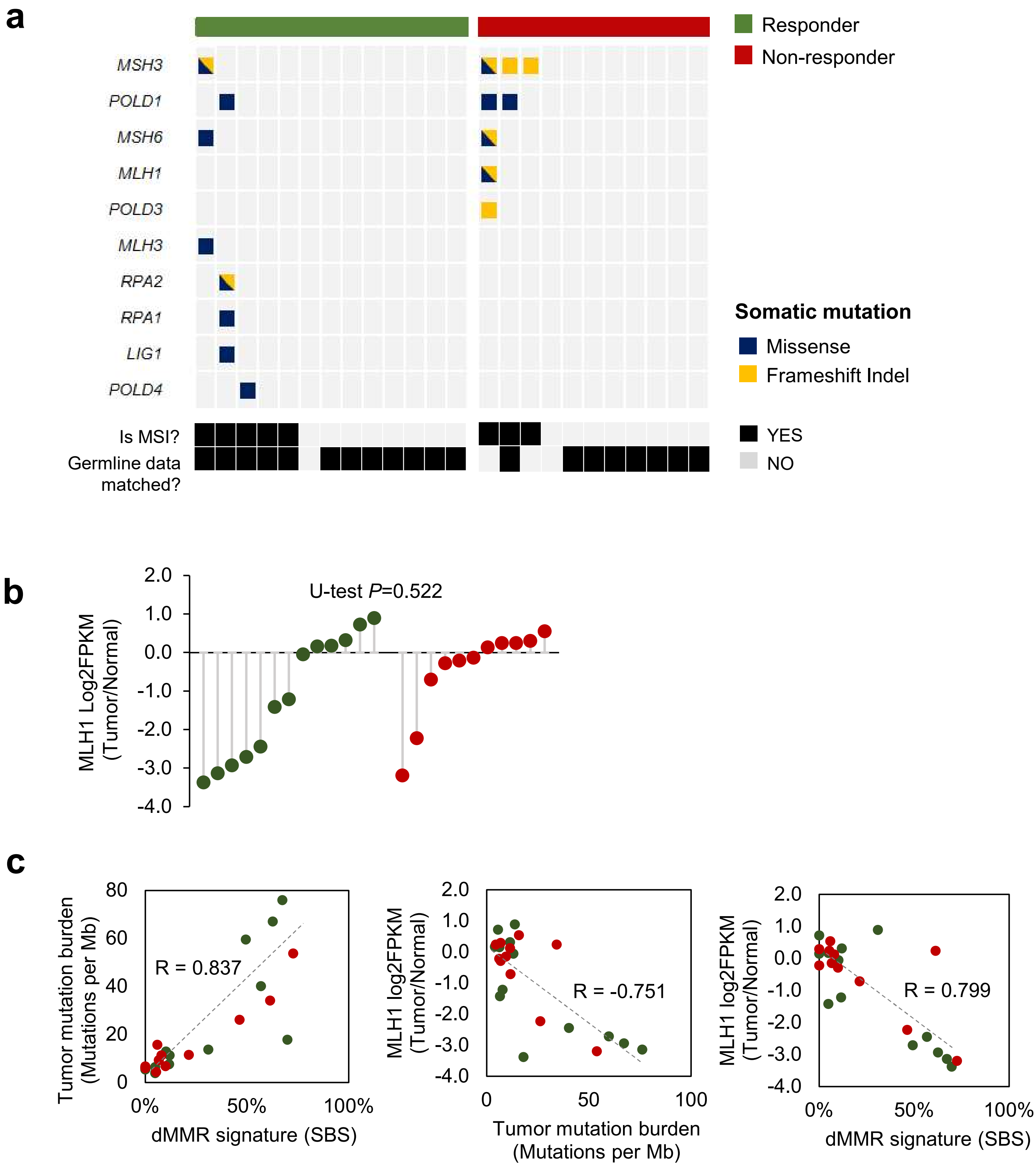

Supplementary Figure 6. Alterations of genes in mismatch repair (MMR) pathway based on KEGG gene-set. a, Mutational profiles of MMR pathway genes in PDX tumors. **b**, Gene expression profiles of *MLH1* in PDX tumors. Two-tailed Mann-whitney U test for P-value. **c.** Correlation of tumor mutation burden, defective MMR signature, and *MLH1* expression.

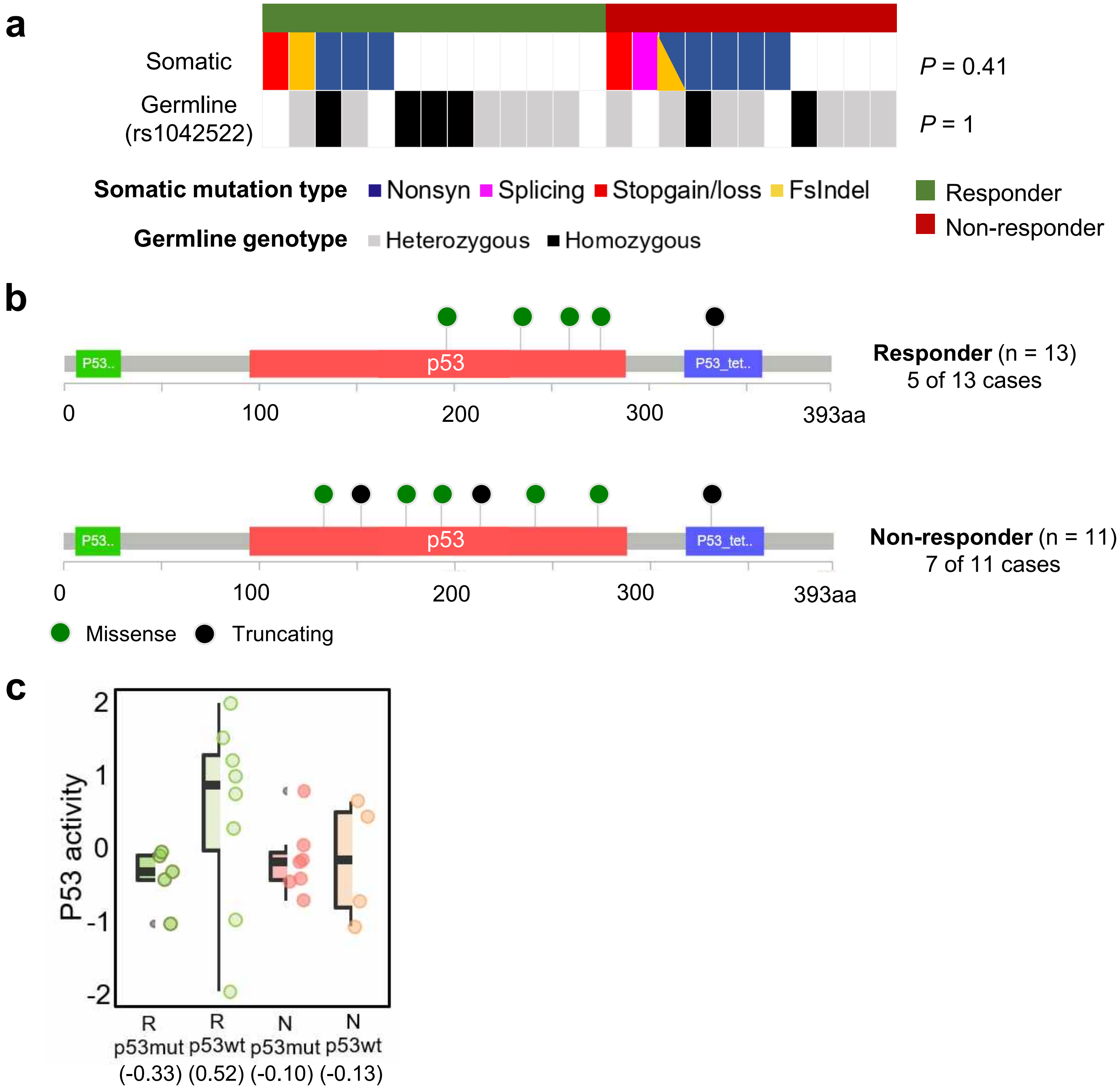

Supplementary Figure 7. Mutational profiles of *TP53* gene and p53-activity signature. a, Overall somatic mutation status and drug-response related germline variant (rs1042522, ClinVar accession: VCV000012351) of *TP53*. Nonsyn: nonsynonymous, FsIndel: frame-shift insertion or deletion. Two-sided Fisher's exact test for P-values. **b,** The p53 protein domains affected by somatic mutations. **c,** p53-activity signature calculated by taking average of FPKM values from *MDM2* and *CDKN1A*, which are p53 transcriptional targets. Non-responder showed low p53 activity regardless of TP53 mutation status. R: responder, N: non-responder.

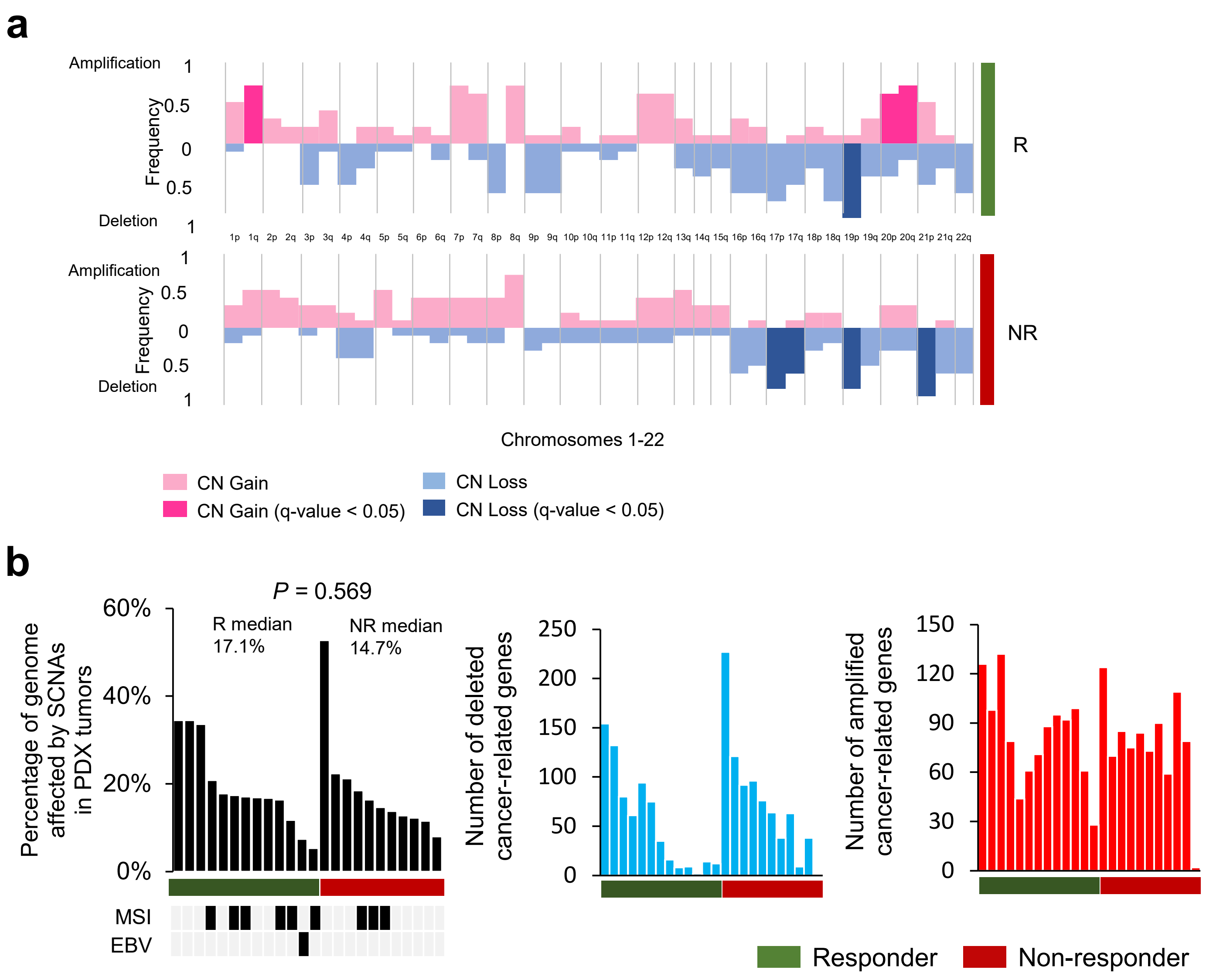

Supplementary Figure 8. Somatic copy number alterations (SCNAs) in PDX tumors. a, SCNAs of PDX tumors in chromosome levels of 13 R and 11 NR, estimated using whole exome sequencing data. False Discovery Rate (FDR) q-values calculated by GISTIC algorithm. **b,** PDX tumor genome affected by SCNAs (left), deletion (middle), and amplification (right) in responder (n=13) and non-responder (n=11) groups. Two-tailed Mann-Whitney U test (*P*=0.5691).

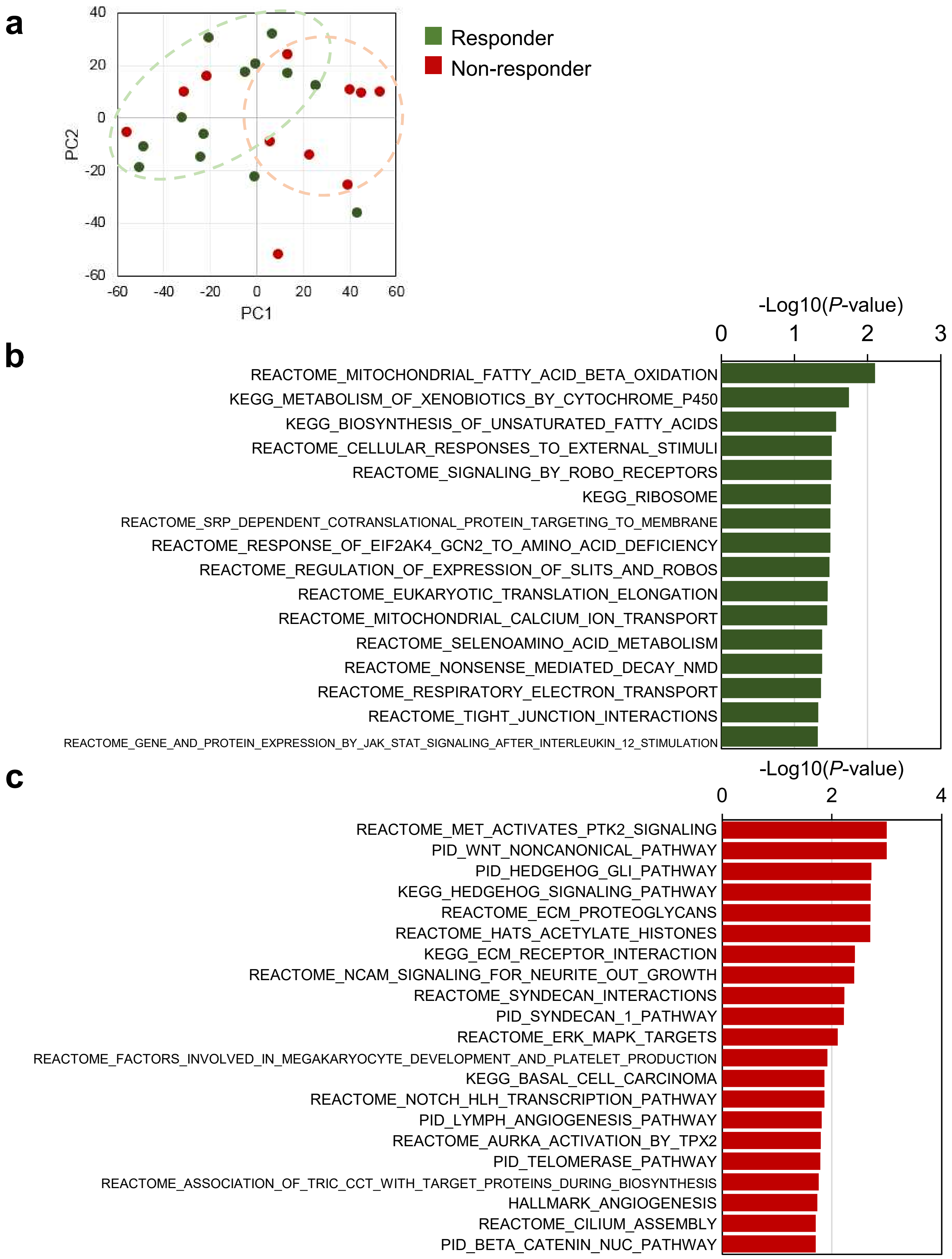
a
Responder
Non-responder
PC1
PC2
b
-Log10(P-value)
REACTOME_MITOCHONDRIAL_FATTY_ACID_BETA_OXIDATION
KEGG_METABOLISM_OF_XENOBIOTICS_BY_CYTOCHROME_P450
KEGG_BIOSYNTHESIS_OF_UNSATURATED_FATTY_ACIDS
REACTOME_CELLULAR_RESPONSES_TO_EXTERNAL_STIMULI
REACTOME_SIGNALING_BY_ROBO_RECEPTORS
KEGG_RIBOSOME
REACTOME_SRP_DEPENDENT_COTRANSLATIONAL_PROTEIN_TARGETING_TO_MEMBRANE
REACTOME_RESPONSE_OF_EIF2AK4_GCN2_TO_AMINO_ACID_DEFICIENCY
REACTOME_REGULATION_OF_EXPRESSION_OF_SLITS_AND_ROBOS
REACTOME_EUKARYOTIC_TRANSLATION_ELONGATION
REACTOME_MITOCHONDRIAL_CALCIUM_ION_TRANSPORT
REACTOME_SELENOAMINO_ACID_METABOLISM
REACTOME_NONSENSE_MEDIATED_DECAY_NMD
REACTOME_RESPIRATORY_ELECTRON_TRANSPORT
REACTOME_TIGHT_JUNCTION_INTERACTIONS
REACTOME_GENE_AND_PROTEIN_EXPRESSION_BY_JAK_STAT_SIGNALING_AFTER_INTERLEUKIN_12_STIMULATION
c
-Log10(P-value)
REACTOME_MET_ACTIVATES_PTK2_SIGNALING
PID_WNT_NONCANONICAL_PATHWAY
PID_HEDGEHOG_GLI_PATHWAY
KEGG_HEDGEHOG_SIGNALING_PATHWAY
REACTOME_ECM_PROTEOGLYCANS
REACTOME_HATS_ACETYLATE_HISTONES
KEGG_ECM_RECEPTOR_INTERACTION
REACTOME_NCAM_SIGNALING_FOR_NEURITE_OUT_GROWTH
REACTOME_SYNDECAN_INTERACTIONS
PID_SYNDECAN_1_PATHWAY
REACTOME_ERK_MAPK_TARGETS
REACTOME_FACTORS_INVOLVED_IN_MEGAKARYOCYTE_DEVELOPMENT_AND_PLATELET_PRODUCTION
KEGG_BASAL_CELL_CARCINOMA
REACTOME_NOTCH_HLH_TRANSCRIPTION_PATHWAY
PID_LYMPH_ANGIOGENESIS_PATHWAY
REACTOME_AURKA_ACTIVATION_BY_TPX2
PID_TELOMERASE_PATHWAY
REACTOME_ASSOCIATION_OF_TRIC_CCT_WITH_TARGET_PROTEINS_DURING_BIOSYNTHESIS
HALLMARK_ANGIOGENESIS
REACTOME_CILIUM_ASSEMBLY
PID_BETA_CATENIN_NUC_PATHWAY

Supplementary Figure 9. Transcriptome analyses of PDX tumors. a, Principal component analysis of RNA sequencing data of PDX tumors from responder and non-responder groups. **b**, Significantly enriched gene sets in significantly up-regulated genes in responder group PDXs compared to non-responder group PDXs. Nominal P-values from GSEA. **c,** Significantly enriched gene sets in significantly up-regulated genes in non-responder group PDXs compared to responder group PDXs. Nominal P-values from GSEA.

a

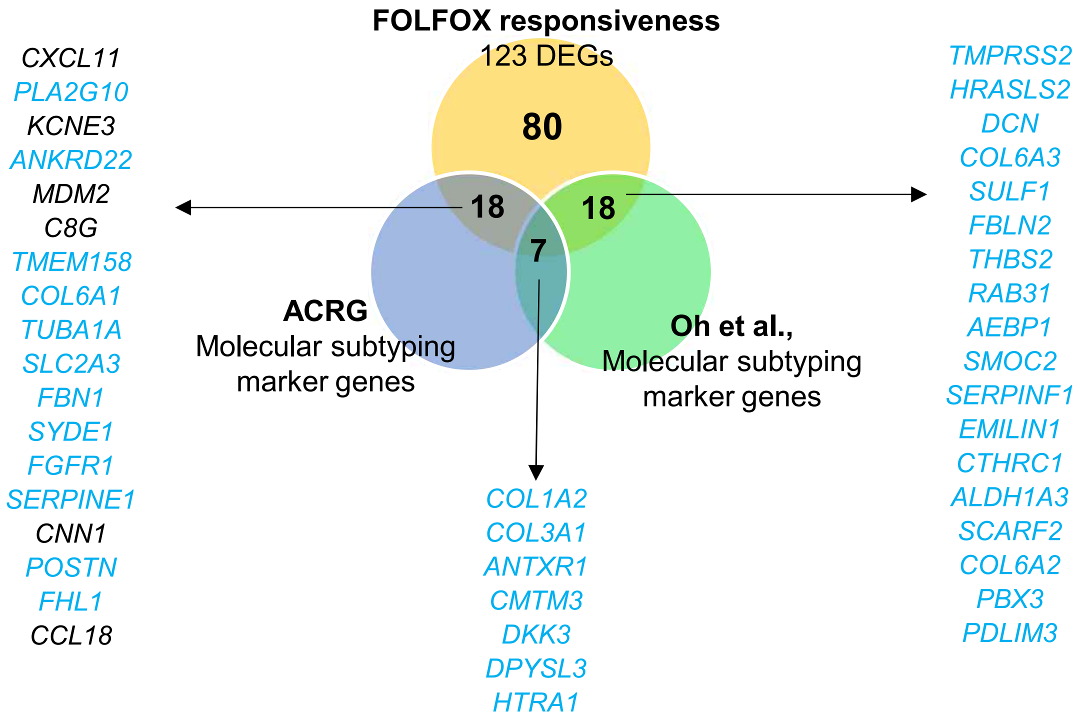

b

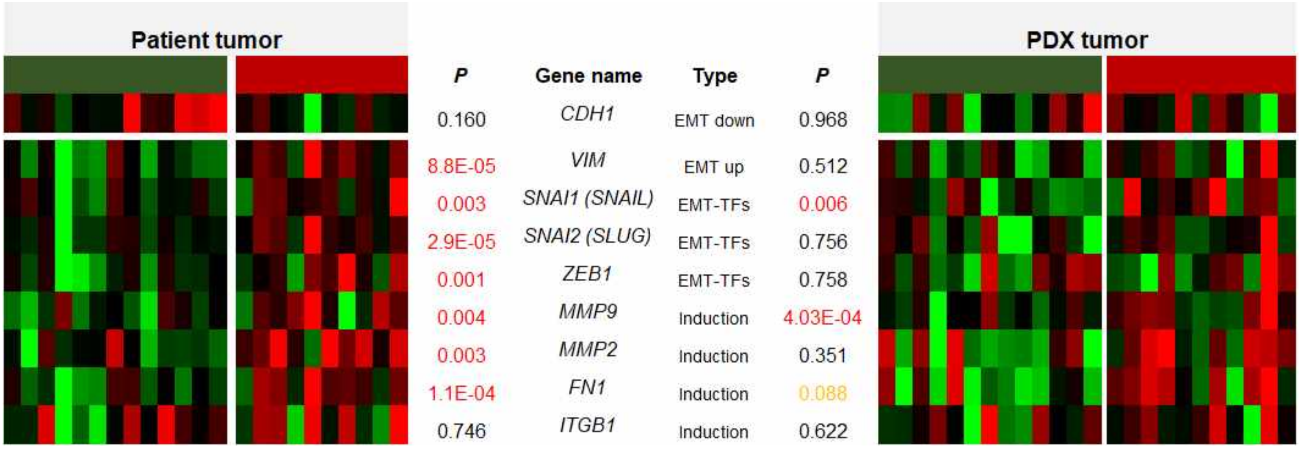

Supplementary Figure 10. Expression of EMT-related genes in PDX models. a, Venn diagram of overlapping genes with previously reported molecular subtyping markers of gastric cancer. Differentially expressed genes between responder and non-responder largely included mesenchymal/EMT-associated genes (genes up-regulated in mesenchymal/EMT are colored in blue). **b,** EMT marker genes are significantly up-regulated in non-responder. EMT-TF: Transcription factor in EMT. Two-sided Wald test for *P*-value.

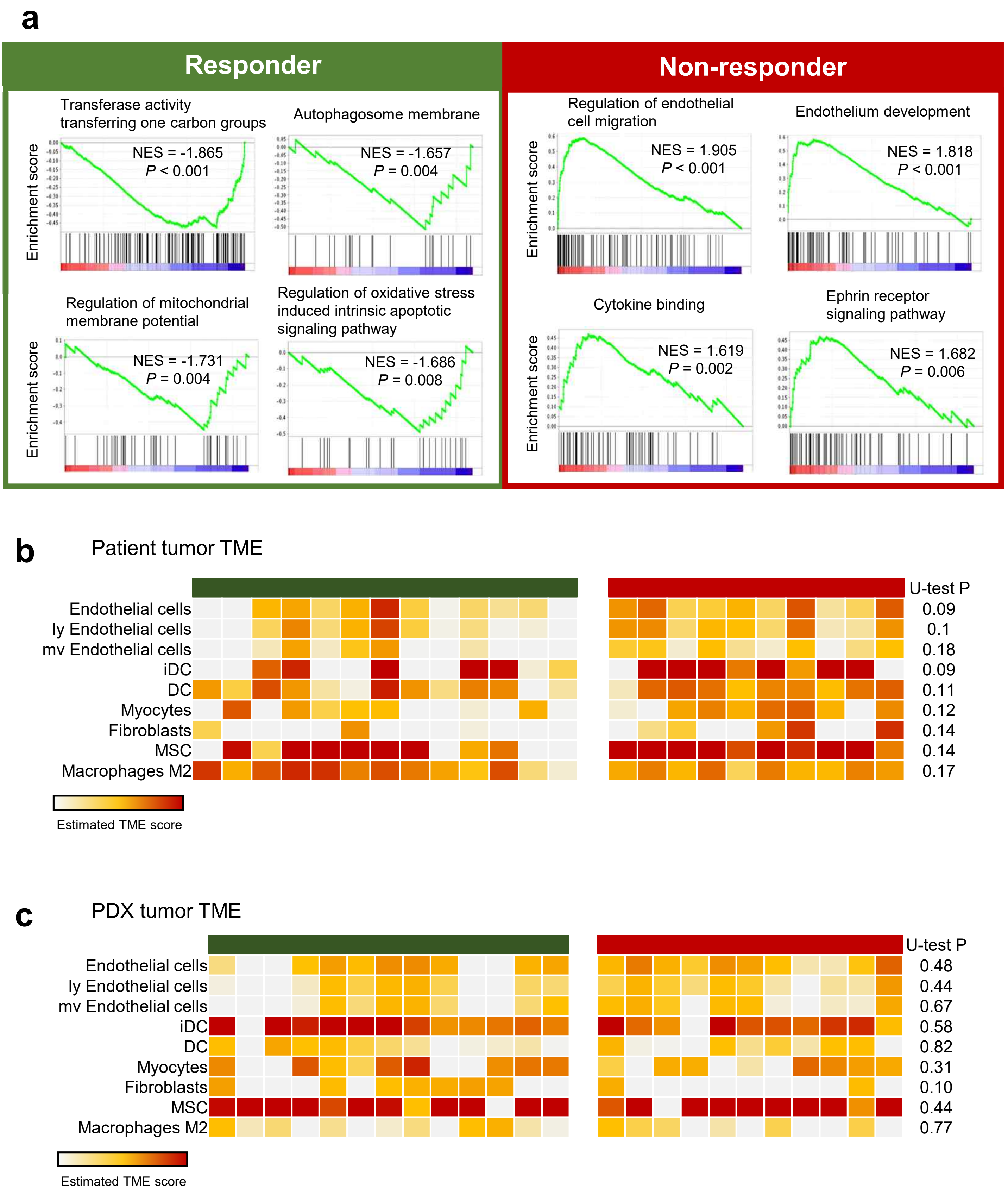
a
Responder
Non-responder
Transferase activity transferring one carbon groups
NES = -1.865
P < 0.001
Autophagosome membrane
NES = -1.657
P = 0.004
Regulation of mitochondrial membrane potential
NES = -1.731
P = 0.004
Regulation of oxidative stress induced intrinsic apoptotic signaling pathway
NES = -1.686
P = 0.008
Regulation of endothelial cell migration
NES = 1.905
P < 0.001
Endothelium development
NES = 1.818
P < 0.001
Cytokine binding
NES = 1.619
P = 0.002
Ephrin receptor signaling pathway
NES = 1.682
P = 0.006
Enrichment score
b
Patient tumor TME
U-test P
Endothelial cells 0.09
ly Endothelial cells 0.1
mv Endothelial cells 0.18
iDC 0.09
DC 0.11
Myocytes 0.12
Fibroblasts 0.14
MSC 0.14
Macrophages M2 0.17
Estimated TME score
c
PDX tumor TME
U-test P
Endothelial cells 0.48
ly Endothelial cells 0.44
mv Endothelial cells 0.67
iDC 0.58
DC 0.82
Myocytes 0.31
Fibroblasts 0.10
MSC 0.44
Macrophages M2 0.77
Estimated TME score

d

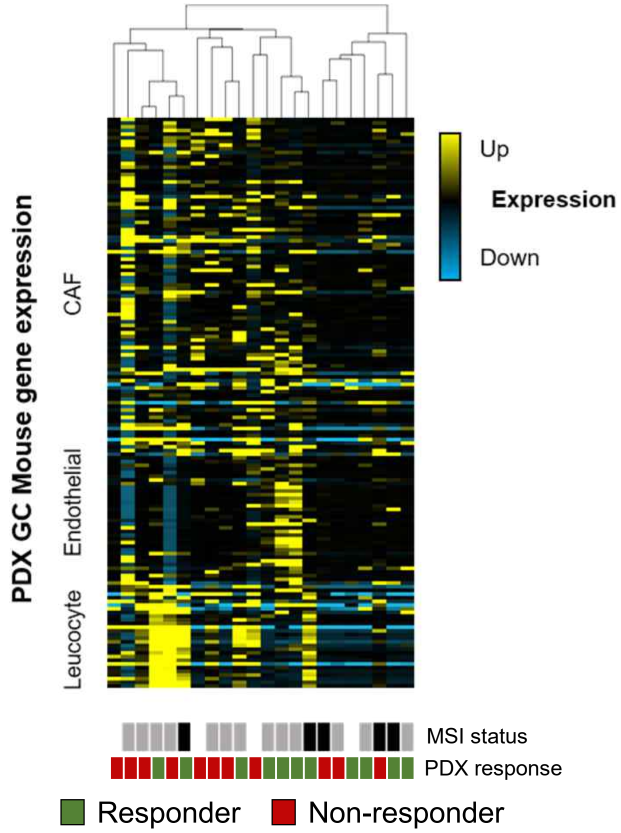

Supplementary Figure 11. Transcriptome analysis of tumor microenvironment in responder and non-responder groups. a, Gene set enrichment analysis of tumor microenvironment inferred by mouse-specific gene expression from PDX tumors, based on Gene Ontology and KEGG terms comparing responder and non-responder. **b,** Composition of stromal cells in patient tumors inferred from RNA sequencing data using xCell algorithm. **c**, Composition of stromal cells in PDX tumors inferred from RNA sequencing data using xCell algorithm. Two-tailed Mann-whitney U test for P values in b-c. **d**, Unsupervised clustering of gene expression in responders and non-responders based on a set of marker genes associated with stromal cells, such as cancer-associated fibroblasts (CAFs), endothelial cells and leucocytes.

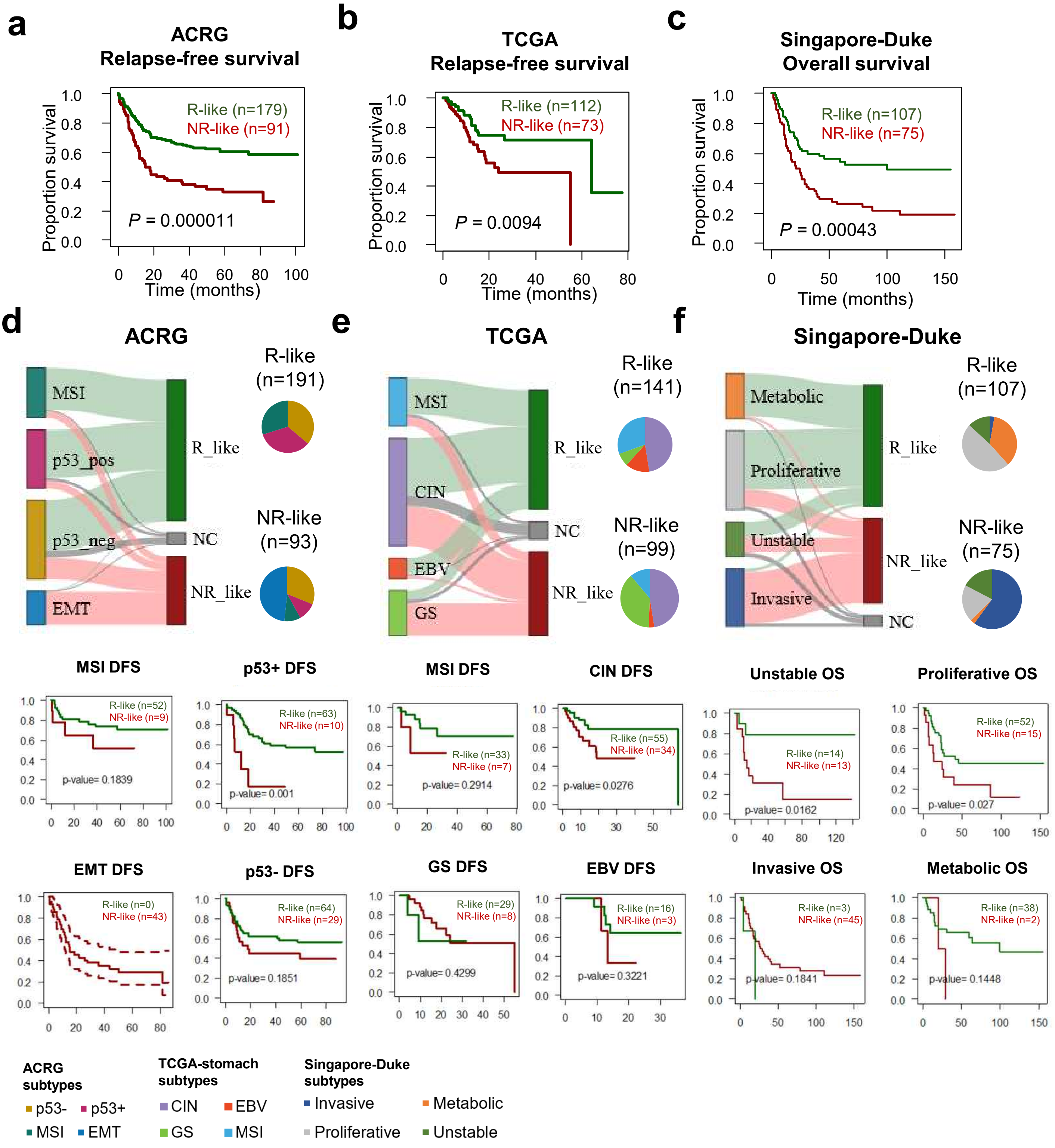

a
ACRG
Relapse-free survival
R-like (n=179)
NR-like (n=91)
P = 0.000011
Proportion survival
Time (months)
b
TCGA
Relapse-free survival
R-like (n=112)
NR-like (n=73)
P = 0.0094
c
Singapore-Duke
Overall survival
R-like (n=107)
NR-like (n=75)
P = 0.00043
d
ACRG
R-like
(n=191)
NR-like
(n=93)
MSI
p53_pos
p53_neg
EMT
R_like
NC
NR_like
e
TCGA
R-like
(n=141)
NR-like
(n=99)
MSI
CIN
EBV
GS
f
Singapore-Duke
R-like
(n=107)
NR-like
(n=75)
Metabolic
Proliferative
Unstable
Invasive
MSI DFS
R-like (n=52)
NR-like (n=9)
p-value= 0.1839
p53+ DFS
R-like (n=63)
NR-like (n=10)
p-value= 0.001
MSI DFS
R-like (n=33)
NR-like (n=7)
p-value= 0.2914
CIN DFS
R-like (n=55)
NR-like (n=34)
p-value= 0.0276
Unstable OS
R-like (n=14)
NR-like (n=13)
p-value= 0.0162
Proliferative OS
R-like (n=52)
NR-like (n=15)
p-value= 0.027
EMT DFS
R-like (n=0)
NR-like (n=43)
p53- DFS
R-like (n=64)
NR-like (n=29)
p-value= 0.1851
GS DFS
R-like (n=29)
NR-like (n=8)
p-value= 0.4299
EBV DFS
R-like (n=16)
NR-like (n=3)
p-value= 0.3221
Invasive OS
R-like (n=3)
NR-like (n=45)
p-value= 0.1841
Metabolic OS
R-like (n=38)
NR-like (n=2)
p-value= 0.1448
ACRG subtypes
p53-
p53+
MSI
EMT
TCGA-stomach subtypes
CIN
EBV
GS
MSI
Singapore-Duke subtypes
Invasive
Metabolic
Proliferative
Unstable

Supplementary Figure 12. Initial 5-FU + oxaliplatin response prediction model developed using 123 DEGs. a-c, Survival analyses for GC patients in ACRG (a), TCGA (b), and Singapore-Duke (c) studies, according to prediction model. Patients were stratified by our prediction model, and the responder-like (R-like) patient group showed a significantly better prognosis than the non-responder-like (NR-like) group with a 123-gene prediction model. P-values were calculated from the log-rank test. ACRG: the Asian Cancer Research Group. **d-f,** Reassignment of GC patients from the ACRG study (d), TCGA (e), and Singapore-Duke (f) studies according to prediction model (Top). Survival analyses for subgroups in ACRG (d), TCGA (e), and Singapore-Duke (f) studies, according to prediction model (bottom). P-values were calculated from the two-sided log-rank test. p53+ group of ACRG cohort, CIN group of TCGA cohort and Unstable and Proliferative groups of Singapore-Duke cohort showed significant differences in survival between R-like and NR-like groups.

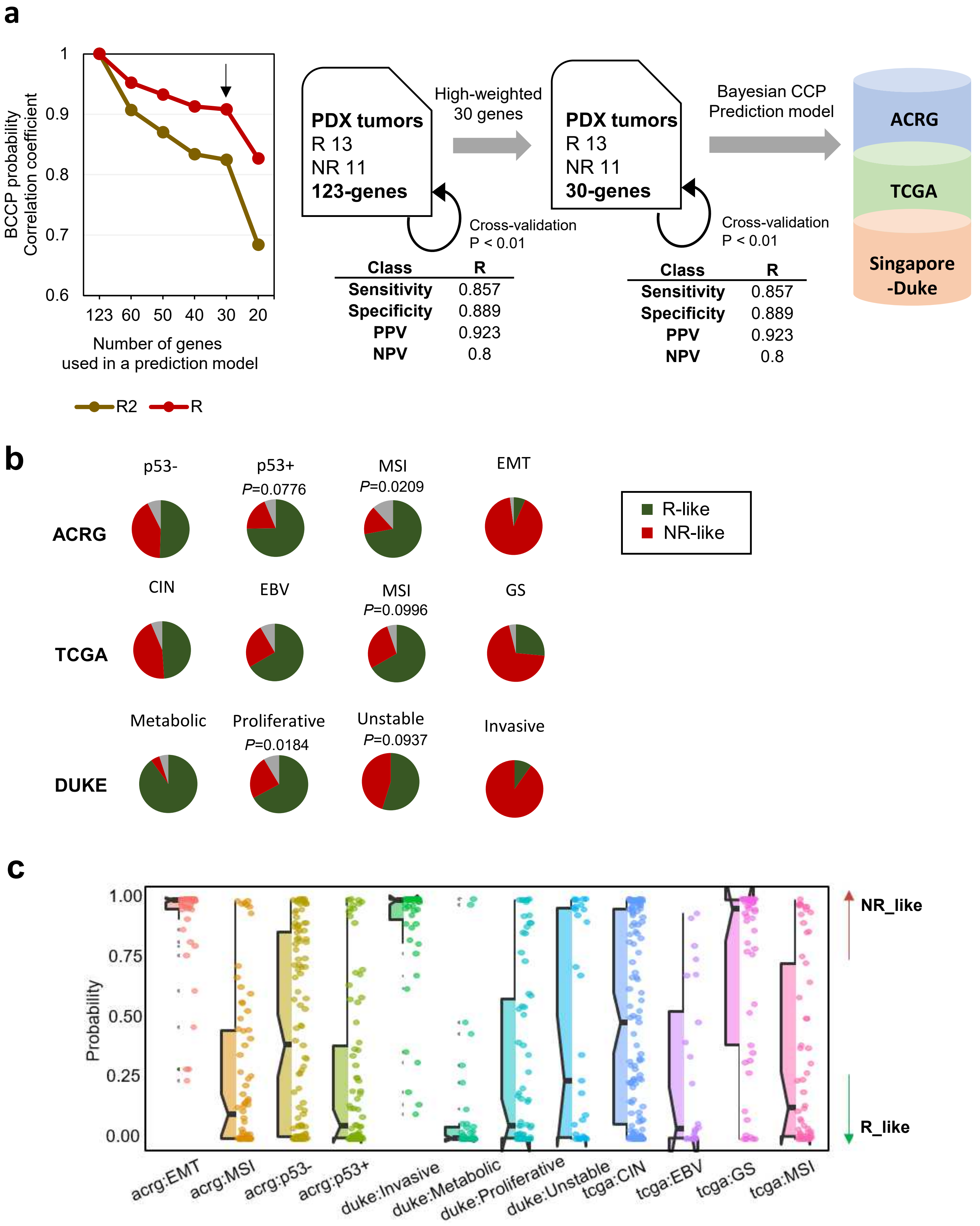
a
BCCP probability Correlation coefficient
1
0.9
0.8
0.7
0.6
123 60 50 40 30 20
Number of genes used in a prediction model
R2
R
PDX tumors
R 13
NR 11
123-genes
High-weighted 30 genes
PDX tumors
R 13
NR 11
30-genes
Bayesian CCP Prediction model
ACRG
TCGA
Singapore -Duke
Cross-validation P < 0.01
Class R
Sensitivity 0.857
Specificity 0.889
PPV 0.923
NPV 0.8
Cross-validation P < 0.01
Class R
Sensitivity 0.857
Specificity 0.889
PPV 0.923
NPV 0.8
b
ACRG
p53-
p53+ P=0.0776
MSI P=0.0209
EMT
TCGA
CIN
EBV
MSI P=0.0996
GS
DUKE
Metabolic
Proliferative P=0.0184
Unstable P=0.0937
Invasive
R-like
NR-like
c
Probability
1.00
0.75
0.50
0.25
0.00
NR_like
R_like
acrg:EMT
acrg:MSI
acrg:p53-
acrg:p53+
duke:Invasive
duke:Metabolic
duke:Proliferative
duke:Unstable
tcga:CIN
tcga:EBV
tcga:GS
tcga:MSI

Supplementary Figure 13. Building a 30-gene 5-FU + oxaliplatin responsiveness prediction model. a, Development of predictive model applying Bayesian CCP (BCCP) algorithm and selection of the minimum number of genes in the predictive model. **b,** The proportion of predicted results of 5-FU + oxaliplatin responsiveness by molecular subtypes based on 30-gene model. Two-sided log-rank test between R-like and NR-like groups for difference in survival. **c,** The BCCP probability of each patient in validation cohorts by molecular subtypes.

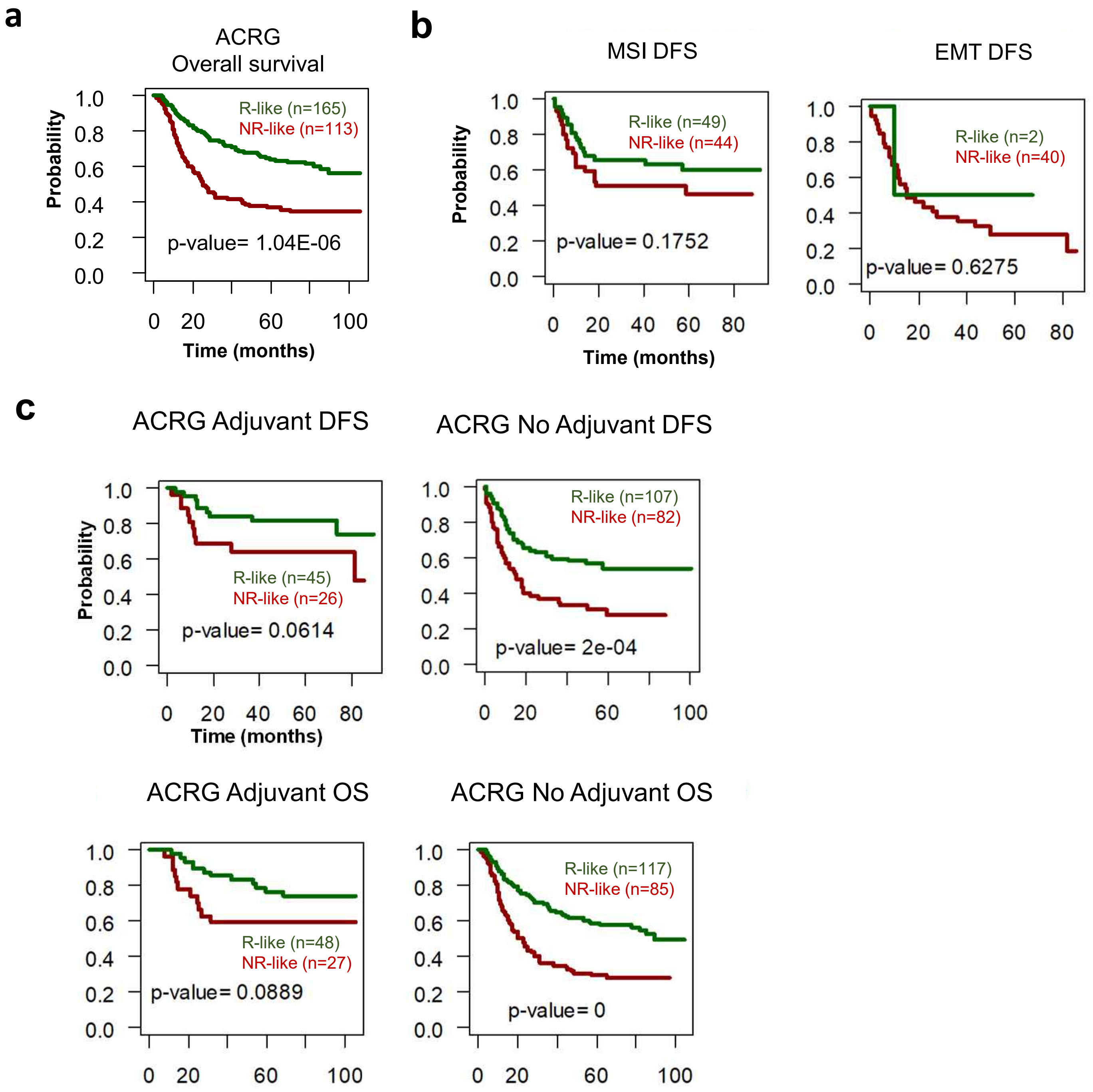

Supplementary Figure 14. Survival analysis of Asian Cancer Research Group (ACRG) GC patients reclassified by our 30-gene response prediction model. a, Patients were stratified by our prediction model in responder-like (R-like) and non-responder-like (NR-like), and overall survival was compared between two groups. **b**, Disease-free survival (DFS) and overall survival (OS) analyses between R-like and NR-like patients in each molecular subtype of ACRG study. **c,** DFS and OS analysis of patients regarding the chemotherapy status. Two-sided log-rank test was performed for *P*-values in (a)-(c).

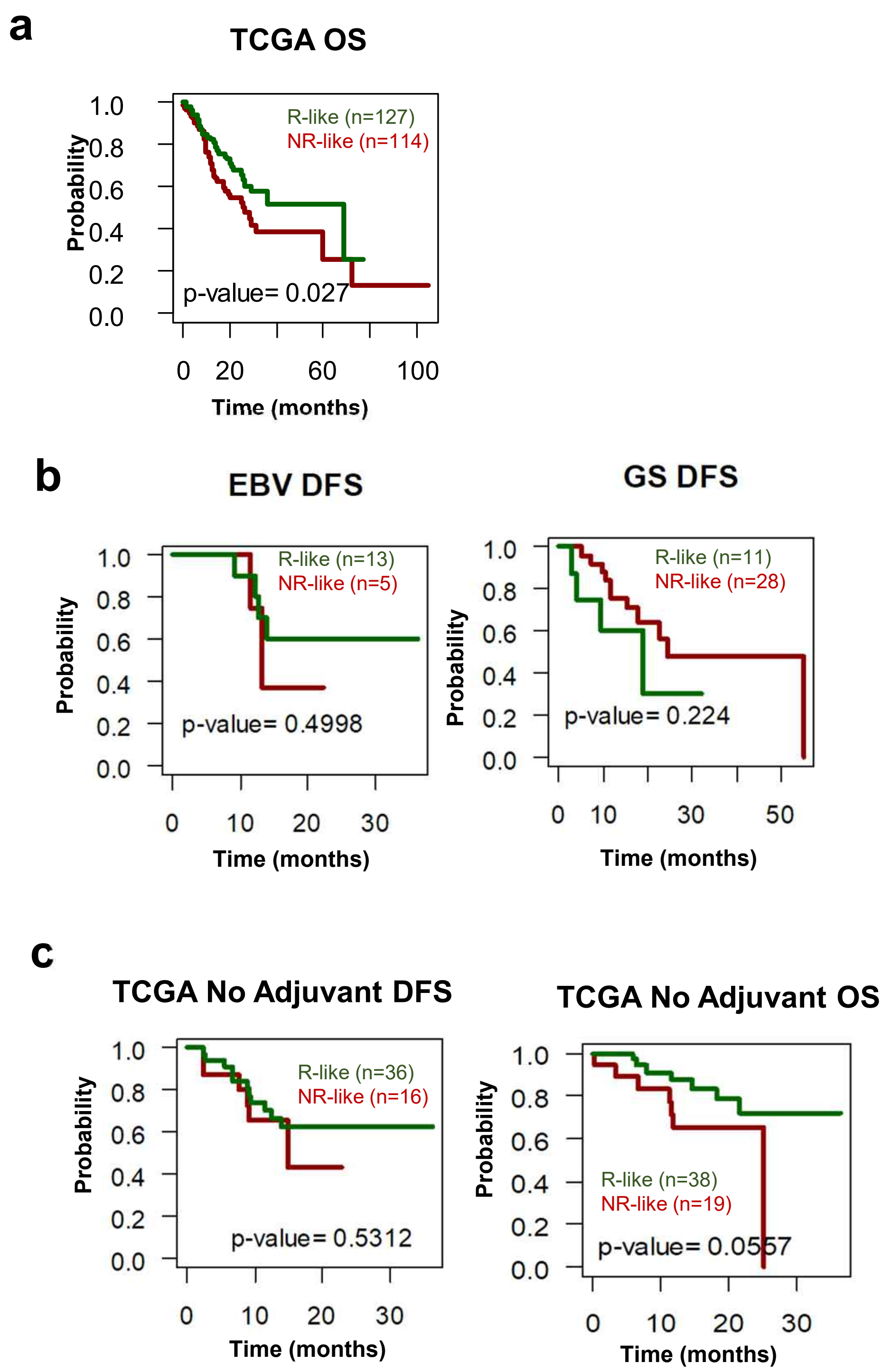

Supplementary Figure 15. Survival analysis of TCGA study GC patients reclassified by our 30-gene response prediction model. a, Patients were stratified by our prediction model as responder-like (R-like) and non-responder-like (NR-like). OS was compared using two-sided log-rank test. **b,** DFS was compared between R-like and NR-like patients by each molecular subtype of TCGA study. Two-sided log-rank test for (b)-(c). **c,** DFS and OS were compared regarding patients' adjuvant chemotherapy status.

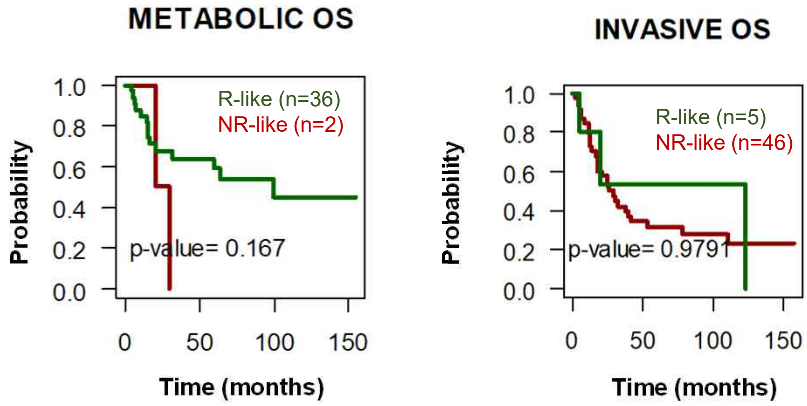

Supplementary Figure 16. Survival analysis of Singapore-Duke study GC patients reclassified by our 30-gene response prediction model. OS of patients stratified by our prediction model were analyzed by molecular subtypes of Singapore-Duke study (Two-sided log-rank test for P-value).

Supplementary table S1. List of 31 patients underwent GC PDX generation and NGS analyses

							WES			WTS			
**Patient #**	**PDX ID**	**MSI status**	**WHO classification**	**Lauren classification**	**TNM stage**	**Location**	**Blood**	**Patient tumor**	**Xenograft tumor**	**Patient nontumor**	**Patient tumor**	**Xenograft tumor**	**Pt_response**
**#1**	SNUH-JAX-G018	MSI-H	PD tub	M	IIIc	lower	v		v	v		v	R
**#2**	SNUH-JAX-G033	MSS	MD tub	I	IIIa	middle	v	v	v	v	v	v	NR
**#3**	SNUH-JAX-G042	MSS	MD tub	I	IIIb	lower	v	v	v	v	v	v	R
**#4**	SNUH-JAX-G064	MSI-H					v	v	v	v	v	v	
**#5**	SNUH-JAX-G067	MSI-H	MD tub	I	IIa		v	v	v	v	v	v	R
**#6**	SNUH-JAX-G080	MSS		M	IIIc	lower	v	v	v	v	v	v	NR
**#7**	SNUH-JAX-G082	MSI-L	PD tub	I	IIIa	lower	v	v	v	v	v	v	
**#8**	SNUH-JAX-G088	MSS	PD tub	M	IIIa	lower	v	v	v	v	v	v	
**#9**	SNUH-JAX-G114	MSS	PCC	D	IIIb	middle	v	v	v	v	v	v	NR
**#10**	SNUH-JAX-G127	MSS		D	IIIc	lower	v	v	v		v	v	NR
**#11**	SNUH-JAX-G129	MSS			IIIc	lower	v	v	v	v	v	v	NR
**#12**	SNUH-JAX-G140	MSI-H	MD tub	I	IIIa	upper		v	v		v	v	
**#13**	SNUH-JAX-G141	MSS	MD tub	I	IV	entire	v	v	v	v	v	v	
**#14**	SNUH-JAX-G170	MSI-H	MD tub	I	IIa	upper	v	v	v		v	v	
**#15**	SNUH-JAX-G182	MSI-H	PD tub	M	Ib	lower	v	v	v	v	v	v	
**#16**	SNUH-JAX-G187	MSS	PD tub	D	IIIb	middle	v	v	v	v	v	v	
**#17**	SNUH-JAX-G209	MSI-H	PD tub	M	Ib	middle	v	v	v		v	v	
	SNUH-JAX-G210						v	v	v		v	v	
**#18**	SNUH-JAX-G213	MSI-H	PD tub	M	IIIc		v	v	v		v	v	
**#19**	SNUH-JAX-G220	MSI-L	PCC		IIIc	lower		v	v		v	v	R
**#20**	SNUH-JAX-G221	MSI-H						v	v		v	v	
**#21**	SNUH-JAX-G222	MSS	PD tub		IIIb	upper	v	v	v	v	v	v	
**#22**	SNUH-JAX-G247	MSS	PCC	D	IIIc	lower	v	v	v	v	v	v	
**#23**	SNUH-JAX-G250	MSS		I	IIIa	lower	v	v	v	v	v	v	NR
**#24**	SNUH-JAX-G263	MSS	PD tub	D	IIb	upper	v	v	v		v	v	
**#25**	SNUH-JAX-G284	MSI-H	MD tub	I	IIIc	lower	v	v	v		v	v	
**#26**	SNUH-JAX-G304	MSS	PD tub	M	IIIb	lower	v	v	v	v	v	v	NR
**#27**	SNUH-JAX-G311	MSS	MD tub	I	IIIc	lower	v	v	v		v	v	
**#28**	SNUH-JAX-G345	MSI-H	PD tub	I		middle	v	v	v			v	
**#29**	SNUH-JAX-G362	MSS	PD tub	D		lower	v	v	v		v	v	
**#30**	SNUH-JAX-G399	MSS	PD tub	M		lower		v	v		v	v	
**#31**	SNUH-JAX-IMX47	MSS					v		v			v	NR

* PD: poorly differentiated, MD: moderlately differentiated, tub: tubular adenocarcinoma, PCC: poorly cohesive carcinoma, I: intestinal, D: diffuse, M: mixed, R: responsive, NR: nonrepor

Supplementary table S2. Result of drug screening and determination of responsiveness in 32 PDX models.

Xenograft ID	N replicates in each treatment and vehicle group	Vehicle Average ± SD	5-FU + oxaliplatin Average ± SD	ANOVA *P*- value	RTV (Treated/Control)	%TGI	PDX response
SJ-G018	4	2074.6 ± 869.6	824.6 ± 915.4	0.005	0.398	0.43	Intermetdiate
SJ-G033	3	524.7 ± 159.8	467.5 ± 163.3	0.715	0.891	0.18	Non-responder
SJ-G042	7	2730.0 ± 1022.7	775.3 ± 452.7	2.24E-23	0.284	0.78	Responder
SJ-G064	3	806.5 ± 235.4	226.6 ± 83.7	7.23E-09	0.281	0.88	Responder
SJ-G067	7	2719.7 ± 668.2	612.3 ± 324.8	2.90E-21	0.225	0.84	Responder
SJ-G080	5	1092.8 ± 189.5	803.1 ± 119.2	8.81E-05	0.735	0.38	Intermetdiate
SJ-G082	4	1353.8 ± 534.9	858.8 ± 369.3	0.026	0.634	0.38	Intermetdiate
SJ-G088	3	650.8 ± 243.0	520.6 ± 373.0	0.986	0.800	0.25	Non-responder
SJ-G114	3	738.5 ± 362.2	520.8 ± 341.6	0.367	0.674	0.33	Non-responder
SJ-G127	4	3490.0 ± 545.2	1103.8 ± 616.1	5.28E-10	0.316	0.72	Responder
SJ-G129	3	2788.8 ± 830.1	1957.5 ± 736.8	0.091	0.702	0.33	Non-responder
SJ-G140	3	554.3 ± 41.1	449.0 ± 135.3	0.915	0.810	0.29	Non-responder
SJ-G141	4	5348.3 ± 3382.8	4204.8 ± 1991.1	0.494	0.786	0.22	Non-responder
SJ-G170	4	520.6 ± 111.4	340.0 ± 46.2	0.002	0.653	0.50	Intermetdiate
SJ-G182	4	1221.8 ± 237.4	493.4 ± 80.1	3.64E-13	0.404	0.79	Responder
SJ-G187	5	758.6 ± 177.7	297.4 ± 93.8	1.70E-07	0.392	0.79	Responder
SJ-G209	4	1103.8 ± 242.5	810.5 ± 261.0	0.005	0.734	0.24	Intermetdiate
SJ-G210	3	716.9 ± 208.6	440.8 ± 71.1	0.025	0.615	0.62	Intermetdiate
SJ-G213	4	1515.7 ± 451.2	572.9 ± 186.5	4.68E-10	0.378	0.70	Responder
SJ-G220	4	1553.3 ± 736.0	131.6 ± 136.4	5.50E-15	0.085	0.96	Responder
SJ-G221	3	742.6 ± 289.6	576.7 ± 148.2	0.123	0.777	0.35	Non-responder
SJ-G222	4	1377.8 ± 527.5	473.0 ± 76.1	4.25E-10	0.343	0.76	Responder
SJ-G247	4	1115.0 ± 177.9	815.1 ± 249.6	2.84E-06	0.731	0.34	Intermetdiate
SJ-G250	3	1050.2 ± 828.4	968.8 ± 250.7	0.921	0.923	0.07	Non-responder
SJ-G263	4	1291.8 ± 468.7	370.8 ± 136.8	8.66E-15	0.287	0.84	Responder
SJ-G284	4	1685.5 ± 559.2	647.0 ± 117.8	7.21E-10	0.384	0.70	Responder
SJ-G304	3	810.6 ± 505.7	366.5 ± 407.0	0.295	0.886	0.17	Non-responder
SJ-G311	4	1664.8 ± 585.6	367.5 ± 120.3	1.51E-08	0.221	0.85	Responder
SJ-G345	4	1103.8 ± 242.5	810.5 ± 261.0	0.005	0.734	0.24	Intermetdiate
SJ-G362	4	2053.3 ± 375.7	354.0 ± 32.3	2.19E-24	0.172	0.92	Responder
SJ-G399	4	1806.5 ± 280.7	1815.0 ± 1114.5	0.342	1.005	-0.01	Non-responder
SJ-IMX47	4	1923.7 ± 1187.3	1342.1 ± 513.8	0.125	0.698	0.35	Non-responder

RTV: Relative Tumor Volume. Relative tumor volume changes. TGI: Tumor Growith inhibition.

Supplementary table S3. The number of responder and non-responder by clinical characteristics.

		Responder	Non-responder	*P* -value
**Gender**	Male	11	9	0.855
	Female	2	2	
**Age (median)**		64	60	0.104
**WHO**	MD tub	4 (36%)	3 (43%)	0.875
	PD tub	6 (55%)	3 (43%)	
	PCC	1 (9%)	1 (14%)	
**Lauren**	Intestinal	4 (40%)	4 (50%)	0.407
	Mixed	2 (20%)	3 (38%)	
	Diffuse	4 (40%)	1 (13%)	
**Location1**	Upper 1/3	2 (20%)	1 (11%)	ND
	Middle 1/3	1 (10%)	2 (22%)	
	Lower 1/3	7 (70%)	5 (56%)	
	Entire	0 (0%)	1 (11%)	
**Location2**	LC	4 (33%)	5 (56%)	0.411
	GC	0 (0%)	0 (0%)	
	AW	1 (8%)	2 (22%)	
	PW	3 (25%)	1 (11%)	
	Circular	4 (33%)	1 (11%)	
**TNM stage**	I and II	3 (27%)	0 (0%)	ND
	IIIa and IIIb	3 (27%)	6 (75%)	
	IIIc and IV	5 (45%)	2 (26%)	

* MD: moderately differentiated, PD: poorly differentiated, tub: Tubular adenocarcinoma, PCC: poorly cohesive carcinoma, LC: lesser curvature, GC: greater curvature, AW: anterior wall, PW: posterior wall, ND: not determined

** *P* -values from the Freeman-Halton extension of the Fisher exact probability test

Supplementary table S4. The number of responder and non-responder by TCGA subtypes

P = 0.464	**EBV**	**MSI**	**CIN**	**GS**	**Sample number**
**Responder**	1 (7.7%)	5 (38.5%)	5 (38.5%)	2 (15.4%)	13
**Non-responder**	0 (0%)	2 (18.2%)	6 (54.5%)	3 (27.3%)	11

* *P*-values from the Freeman-Halton extension of the Fisher exact probability test

Supplementary table S5. Clinical characteristics of GC patients of ACRG, Singapore and TCGA cohorts re-classified by 5-FU response 30-gene prediction model

a, ACRG

		R-like (n=165)	N-like (n=113)	NC (n=22)	R-like (n=165)	N-like (n=113)	NC (n=22)
**Age**	Average	61.9	61.7	63.4			
**Sex**	Male	114	69	16	69.1%	61.1%	72.7%
	Female	51	44	6	30.9%	38.9%	27.3%
**Lauren**	Diffuse	58	66	10	35.2%	58.4%	45.5%
	Intestinal	94	43	9	57.0%	38.1%	40.9%
	Mixed	11	3	3	6.7%	2.7%	13.6%
	NA	0	0	0	0.0%	0.0%	0.0%
**pStage**	I	18	10	2	10.9%	8.8%	9.1%
	II	62	27	7	37.6%	23.9%	31.8%
	III	51	38	6	30.9%	33.6%	27.3%
	IV	32	38	7	19.4%	33.6%	31.8%
**WHO**	Poorly differenciated	66	37	13	40.0%	32.7%	59.1%
	Well or moderately differenciated	74	34	6	44.8%	30.1%	27.3%
	Papillary	4	3	2	2.4%	2.7%	9.1%
	Signet	10	26	1	6.1%	23.0%	4.5%
	Hepatoid	1	1	0	0.6%	0.9%	0.0%
	Mucinous	4	4	0	2.4%	3.5%	0.0%
	Others	6	8	0	3.6%	7.1%	0.0%
**Location**	Antrum	83	60	12	50.3%	53.1%	54.5%
	Body	60	38	9	36.4%	33.6%	40.9%
	Cardia	19	12	1	11.5%	10.6%	4.5%
	Whole	3	3	0	1.8%	2.7%	0.0%

b, TCGA-Nature cohort

		R-like (n=131)	N-like (n=116)	NC (n=15)	R-like (n=131)	N-like (n=116)	NC (n=15)
**Age**	Average	66.2	64.3	71.4			
**Sex**	Male	85	71	9	64.9%	61.2%	60.0%
	Female	46	45	6	35.1%	38.8%	40.0%
**Lauren**	Stomach Adenocarcinoma	78	68	12	59.5%	58.6%	80.0%
	Diffuse Type Stomach Adenocarcinoma	15	33	1	11.5%	28.4%	6.7%
	Tubular Stomach Adenocarcinoma	29	4	1	22.1%	3.4%	6.7%
	Mucinous Stomach Adenocarcinoma	5	8	1	3.8%	6.9%	6.7%
	Papillary Stomach Adenocarcinoma	4	1	0	3.1%	0.9%	0.0%
	Signet Ring Cell Carcinoma of the Stomach	0	2	0	0.0%	1.7%	0.0%
**Patient Primary Tumor Site**	Stomach (NOS)	3	1	0	2.3%	0.9%	0.0%
	Gastroesophageal Junction	9	4	1	6.9%	3.4%	6.7%
	Fundus/Body	50	43	5	38.2%	37.1%	33.3%
	Cardia/Proximal	19	12	4	14.5%	10.3%	26.7%
	Antrum/Distal	46	52	5	35.1%	44.8%	33.3%
	NA	4	4	0	3.1%	3.4%	0.0%
**AJCC Tumor Stage Code**	T1 , T1a, T1b	10	1	0	7.6%	0.9%	0.0%
	T2, T2a, T2b	38	23	5	29.0%	19.8%	33.3%
	T3	51	47	6	38.9%	40.5%	40.0%
	T4	2	11	3	1.5%	9.5%	20.0%
	T4a	17	19	0	13.0%	16.4%	0.0%
	T4b	9	10	1	6.9%	8.6%	6.7%
	TX	4	5	0	3.1%	4.3%	0.0%

c, Singapore-Duke

		R-like (n=105)	N-like (n=79)	NC (n=8)	R-like (n=105)	N-like (n=79)	NC (n=8)
**Age**	Average	64.8	63.9	63.2			
**Sex**	Male	70	49	6	66.7%	62.0%	75.0%
	Female	35	30	2	33.3%	38.0%	25.0%
**Lauren classification**	Diffuse	26	46	3	24.8%	58.2%	37.5%
	Intestinal	68	28	3	64.8%	35.4%	37.5%
	Mixed	11	5	2	10.5%	6.3%	25.0%
**Stage**	I	20	9	2	19.0%	11.4%	25.0%
	II	18	11	0	17.1%	13.9%	0.0%
	III	38	29	5	36.2%	36.7%	62.5%
	IV	29	30	1	27.6%	38.0%	12.5%
**Outcome**	0	68	24	5	64.8%	30.4%	62.5%
	1	37	55	3	35.2%	69.6%	37.5%
