# Supplementary material for: Predictive biomarkers for 5-fluorouracil and oxaliplatin-based chemotherapy in gastric cancers via profiling of patient-derived xenografts
Source: Nat Commun. 2021 Aug 10;12:4840. doi: 10.1038/s41467-021-25122-4 (PMC8355375; doi:10.1038/s41467-021-25122-4)
Supplement: Supplementary file 3 — Description of Additional Supplementary Files [file 41467_2021_25122_MOESM3_ESM.docx]

Description of Additional Supplementary Files

Title: Supplementary Dataset 1.

Description: Profile of somatic mutations in oncogenes determined based on OncoKB. Mutations shared in matched patient and PDX tumors are colored in black. PDX tumor-only: blue. Patient tumor-only: orange.

Title: Supplementary Dataset 2.

Description: List of 123 differentially expressed genes between responders and non-responders used to develop a responsiveness prediction model.
