# Supplementary material for: Predictive biomarkers for 5-fluorouracil and oxaliplatin-based chemotherapy in gastric cancers via profiling of patient-derived xenografts
Source: Nat Commun. 2021 Aug 10;12:4840. doi: 10.1038/s41467-021-25122-4 (PMC8355375; doi:10.1038/s41467-021-25122-4)
Supplement: Supplementary file 6 — Reporting Summary [file 41467_2021_25122_MOESM6_ESM.pdf]

nature research

Corresponding author(s): Seock-Ah Im, Han-Kwang Yang, Charles Lee, and Jong-Il Kim

Last updated by author(s): Jun 16, 2021

Reporting Summary

Nature Research wishes to improve the reproducibility of the work that we publish. This form provides structure for consistency and transparency in reporting. For further information on Nature Research policies, see our Editorial Policies and the Editorial Policy Checklist.

Statistics

For all statistical analyses, confirm that the following items are present in the figure legend, table legend, main text, or Methods section.

n/a	Confirmed	
☐	☒	The exact sample size (*n*) for each experimental group/condition, given as a discrete number and unit of measurement
☐	☒	A statement on whether measurements were taken from distinct samples or whether the same sample was measured repeatedly
☐	☒	The statistical test(s) used AND whether they are one- or two-sided<br>*Only common tests should be described solely by name; describe more complex techniques in the Methods section.*
☐	☒	A description of all covariates tested
☐	☒	A description of any assumptions or corrections, such as tests of normality and adjustment for multiple comparisons
☐	☒	A full description of the statistical parameters including central tendency (e.g. means) or other basic estimates (e.g. regression coefficient) AND variation (e.g. standard deviation) or associated estimates of uncertainty (e.g. confidence intervals)
☐	☒	For null hypothesis testing, the test statistic (e.g. *F*, *t*, *r*) with confidence intervals, effect sizes, degrees of freedom and *P* value noted<br>*Give P values as exact values whenever suitable.*
☐	☒	For Bayesian analysis, information on the choice of priors and Markov chain Monte Carlo settings
☐	☒	For hierarchical and complex designs, identification of the appropriate level for tests and full reporting of outcomes
☐	☒	Estimates of effect sizes (e.g. Cohen's *d*, Pearson's *r*), indicating how they were calculated

Our web collection on statistics for biologists contains articles on many of the points above.

Software and code

Policy information about availability of computer code

Data collection	Whole exome sequencing (WES) and whole transcriptome sequencing (WTS) was performed on Illumina HiSeq 2000 instruments.
Data analysis	WES align: BWA mem version 0.7.10<br>WTS align: STAR aligner version 2.4.0i<br>Picard tools: version 1.124<br>GATK version 3.2<br>MuTect version 1.1.7 and Indelocator version 36.3336<br>Annovar version 2017Jul16<br>deconstructSigs version 1.6.0<br>PyClone version 0.13.0<br>CoNIFER version 0.2.2 and DNAcopy version 1.50.1<br>edgeR version 3.12.1<br>GenePattern version 3.9.0<br>DESeq2 version 1.10.1<br>DAVID genome analysis version 6.8<br>STRING database version 10.0<br>xCell version 1.12<br>SPSS software version 22.0<br>BRB-ArrayTools version 4.5.0

For manuscripts utilizing custom algorithms or software that are central to the research but not yet described in published literature, software must be made available to editors and reviewers. We strongly encourage code deposition in a community repository (e.g. GitHub). See the Nature Research guidelines for submitting code & software for further information.

Data

Policy information about availability of data

All manuscripts must include a data availability statement. This statement should provide the following information, where applicable:

- Accession codes, unique identifiers, or web links for publicly available datasets
- A list of figures that have associated raw data
- A description of any restrictions on data availability

The PDX sequencing data generated in this study have been deposited in the European Nucleotide Archive (ENA) repository under accession code PRJEB40936 [https://www.ebi.ac.uk/ena/browser/view/PRJEB40936]. Source data are provided with this paper. The TCGA validation data used in this study are available in the FIREHOSE database [http://firebrowse.org/?cohort=STAD]. The ACRG expression data used in this study are available in the Gene Expression Omnibus (GEO) database under accession code GSE62254 [https://www.ncbi.nlm.nih.gov/geo/query/acc.cgi?acc=GSE62254]. The Singapore-Duke expression data used in this study are available in the GEO database under accession code GSE15459 [https://www.ncbi.nlm.nih.gov/geo/query/acc.cgi?acc=GSE15459]

Field-specific reporting

Please select the one below that is the best fit for your research. If you are not sure, read the appropriate sections before making your selection.

☒ Life sciences ☐ Behavioural & social sciences ☐ Ecological, evolutionary & environmental sciences

For a reference copy of the document with all sections, see nature.com/documents/nr-reporting-summary-flat.pdf

Life sciences study design

All studies must disclose on these points even when the disclosure is negative.

Sample size	We established a GC PDX cohort consisting of total 32 PDX cases from 31 GC patients. The sample size was determined basing on the recruitment capacity of the study as well as on the in vivo PDX tumor availability. Total 32 PDX models were classified into 13 responders (R), 11 non-responders (NR), and 8 cases with questionable responsiveness. Multiple independent measurements were taken for each PDX to determine drug responsiveness. Sample size for the performed assays was optimized based on expected mean differences and according to assay manufacturer's indications (where applicable) and on previous experiments.
Data exclusions	no data exclusions
Replication	Experimental findings were reliably reproduced as described in manuscript and figure legends.
Randomization	Mice included in the in vivo experiment were randomized by body weight and tumor size after the tumors reached approximately 200 mm3 and were divided into treatment and control groups (N=5 each).
Blinding	Blinding was not performed. Due to the chemical nature of the compounds and their schedule of administration, blinding during treatment administration was not feasible.

Reporting for specific materials, systems and methods

We require information from authors about some types of materials, experimental systems and methods used in many studies. Here, indicate whether each material, system or method listed is relevant to your study. If you are not sure if a list item applies to your research, read the appropriate section before selecting a response.

Materials & experimental systems

n/a	Involved in the study	
☒	☐	Antibodies
☒	☐	Eukaryotic cell lines
☒	☐	Palaeontology and archaeology
☐	☒	Animals and other organisms
☐	☒	Human research participants
☒	☐	Clinical data
☒	☐	Dual use research of concern

Methods

n/a	Involved in the study	
☒	☐	ChIP-seq
☒	☐	Flow cytometry
☒	☐	MRI-based neuroimaging

Animals and other organisms

Policy information about studies involving animals; ARRIVE guidelines recommended for reporting animal research

Laboratory animals	NOD/SCID/IL-2γ-receptor null (NSG mice, The Jackson Laboratory, 4-weeks old) female mice

Wild animals	No wild animals were used in this study.
Field-collected samples	No field-collected samples were used in this study.
Ethics oversight	Mice were cared for according to institutional guidelines of the Institutional Animal Care and Use Committee (IACUC) of Seoul National University Hospital (No. 14-0016-C0A0).

Note that full information on the approval of the study protocol must also be provided in the manuscript.

Human research participants

Policy information about studies involving human research participants

Population characteristics	Gastric cancer patients who underwent gastrectomy at Seoul National University Hospital from 2014 to 2017 consisting of 25 males and 6 females. The mean age of patients was 63.3 years old (range 40 - 86), which was included in Supplementary Table 1.
Recruitment	Gastric cancer patients who underwent gastrectomy at Seoul National University Hospital from 2014 to 2017 were recruited. Patients who were treated preoperatively with chemotherapy or chemoradiation therapy were not included. Selection bias was limited as much as possible, but the participants were recruited on a single-center basis for a population of Korean.
Ethics oversight	This study was approved by the institutional review board of the Seoul National University Hospital (No. C-1402-054-555) in accordance with the Declaration of Helsinki.

Note that full information on the approval of the study protocol must also be provided in the manuscript.
